# MYSTETH—home-based heart monitoring

**DOI:** 10.3389/fdgth.2025.1616334

**Published:** 2025-09-30

**Authors:** Kopal Jain, Rohit Jain, Salik Khwaja Mohammad, Swati Aggarwal

**Affiliations:** ^1^ Department of Electrical Engineering, Indian Institute of Technology, Kharagpur, West Bengal, India; ^2^ Department of Computer Science and Engineering, Netaji Subhas University of Technology, New Delhi, India; ^3^ Faculty of Logistics, Molde University College, Molde, Norway

**Keywords:** cardiac disease screening, heart sounds, murmurs, systolic murmurs, diastolic murmurs

## Abstract

**Introduction:**

The MySteth is an intelligent medical tool designed for cardiac disease screening, utilizing either a stethoscope or smartphone to record heart sounds. Normal heart sounds in healthy individuals consist of “lub” and “dub” noises, while murmurs—additional sounds during heartbeats—can indicate cardiac anomalies such as valve dysfunctions and rapid blood flow, categorized as systolic or diastolic.

**Method:**

MySteth was developed and tested using heart sounds recorded via smartphone and digital stethoscope. For ensuring the clinical validity of the data, all heart sound samples were meticulously annotated by human experts—super-specialized cardiologists with extensive experience in cardiac diagnostics. To achieve high classification accuracy, MySteth employs a hybrid CNN-LSTM model combined with Linear Predictive Coding (LPC) for preprocessing. The study involves classifying recorded heart sounds into normal heartbeats and murmurs, with murmurs further divided into systolic and diastolic categories.

**Results:**

The tool demonstrated an accuracy of 92% in distinguishing normal heartbeats from murmurs, 91% in classifying murmurs into systolic and diastolic types, and 90% in further categorizing systolic murmurs into Ejection Systolic Murmurs (ESM) and Pansystolic Murmurs (PSM). MySteth is accessible and affordable, requiring minimal equipment, as most individuals already own a smartphone, and digital stethoscopes are commonly available. This ease of use facilitates both professional and home-based heart monitoring, especially beneficial in remote areas with limited healthcare access.

**Discussion:**

MySteth is an at-home heart diagnostic tool that leverages deep learning to classify heart sounds into normal, ESM, PSM, and diastolic murmurs. Its user-friendly design and minimal hardware requirements ensure broad adoption across various healthcare settings, facilitating timely and accurate preliminary heart investigations. This capability is crucial in combating the global burden of cardiovascular diseases. MySteth's scalability and ease of deployment underscore its potential in early cardiovascular disease diagnosis, particularly in underserved regions, thereby promoting preventive healthcare.

## 1 Introduction

The two typical heart sounds in healthy people are a lub and a dub, which happen one after the other with each beating. It's common to refer to the lub as the first heart sound (S1) and a dub as the second heart sound (S2). Additional noises are heard in regular heart sounds (HS), which can be used in pathology diagnosis in circumstances when the heart is aberrant, such as valve dysfunctions and fast blood flow (1). These extra noises, sometimes referred to as murmurs, exhibit distinct traits in relation to heart valve problems, which are circulatory heart illnesses (2). The most common way to categorize cardiac murmurs is by timing; they can be classified as either systolic (3) or diastolic (4), depending on which portion of the heartbeat they occur during.

Murmurs of the heart that are audible during systole are known as systolic murmurs. The most common systolic murmur (5):

1. Ejection-systolic murmurs (ESM): Diamond-shaped or spindle-shaped. The intensity first increases and then decreases during S1.
2. Pansystolic murmurs (PSM): Rectangular shaped. The intensity remains constant during S1.

The murmur heard in the heart during diastole is called diastolic heart murmur. Diastolic murmurs end at or before S1 and begin at or after S2 (6).

Heart murmurs are a problem that affects a large percentage of people worldwide. These murmurs might be an indicator of underlying cardiovascular disorders including valve dysfunctions. About 2.5% of Americans have heart valve disease, with the prevalence rising with age, according to the American Heart Association (7). One of the primary reasons heart murmurs are not timely diagnosed is the lack of access to regular and comprehensive cardiac evaluations, particularly in underserved and rural areas (8). Additionally, the subtle nature of some murmurs can make them difficult to detect without specialized equipment and expertise. The introduction of a home-based preliminary diagnostic tool for heart murmurs could be highly beneficial. Such a tool would enable individuals to monitor their heart sounds regularly, facilitating early detection of abnormalities and prompting timely medical consultations. This proactive approach could significantly reduce the burden of undiagnosed heart conditions, improve patient outcomes, and decrease healthcare costs associated with advanced cardiovascular diseases (9).

With 17.9 million deaths from cardiovascular diseases (CVDs) per year, or 31% of all fatalities globally, CVD is a major public health concern (10). Early detection is key since cardiac disorders can worsen over time and necessitate more involved forms of care. For instance, coronary heart disease, one of the most common cardiac conditions in the United States, can worsen over time and eventually necessitate coronary artery bypass grafting (CABG) (11). Preventative detection of heart diseases is essential, and medical professionals often start by checking the patient's heartbeat and abnormalities. Further tests, such as blood pressure and fasting protein profile tests, are then performed for further analysis (12). Currently, there is no easy method for heart screening at home without specialized medical personnel. Heart health monitoring and the availability of at-home testing options is crucial for promoting heart health awareness. At-home diagnostics can significantly contribute to heart health promotion and better outcomes for those at risk of cardiovascular problems by enabling individuals to adopt proactive measures towards lowering their risk of heart disease (13).

Numerous studies have employed machine learning and deep learning techniques to categorize heartbeat sounds; most of these studies have focused on data from phonocardiography (PCG), a specialized device used for medical diagnostics (14–19). However, this technology is not accessible to the average consumer and cannot be performed at home.

Advancements in technology have led to smartphone applications like SensiCardiac (20), Mobile Stethoscope (21), and iStethoscope Pro (22), which allow heart sounds to be conveniently recorded. Some studies have also used Electrocardiogram (ECG) signals, which are obtained from specialized instruments in medical facilities. Some studies have also used audio from electronic stethoscopes and mobile phones (23). Only a few categories have been used to categorize murmurs: artifact, extra-heart sound, extrasystole, murmur, normal heartbeat, moderate, severe, or normal, aberrant. There has never been an attempt to further categorize murmurs into systolic, diastolic, and systolic murmurs as well as ESM and PSM. Because there aren't enough datasets available, the majority of these studies have limitations (24). The models are trained and validated on specific datasets which may not encompass the full variability seen in global populations. Without prior patient information, other classifications of murmurs—such as mitral valve prolapse, mitral regurgitation, and aortic stenosis—cannot be made. These classifications require further tests such as ECG, ultrasound, and cardiac CT (25). The key to reducing healthcare costs from CVD and increasing patient outcomes lies in early detection, prevention, and access to quality health services (26). Unfortunately, emergency rooms and hospitals are overcrowded, while affordable healthcare clinics are scarce. This created the need for the development of in-home health monitoring and CVD management programs (27). Early detection and prevention are crucial because CVD accounts for 17.9 million deaths yearly (28). Medical technology has advanced, but there are still no easily available, user-friendly techniques for doing at-home cardiac screenings (29). This study looks at how Mysteth uses digital stethoscope technology and deep learning methods to offer a quick and easy way to do initial cardiac investigations. The goal is to raise awareness of heart health issues and improve the lives of people who are at risk of CVDs.

This work presents MySteth as an innovative at-home heart diagnostic tool designed to bridge the gap in care by providing a convenient and accessible solution for preliminary heart investigations. While other heart testing options exist, MySteth offers distinct advantages. It is the first screening method of its kind to use deep learning techniques and recorded heartbeat sounds to detect a wide range of heart diseases using a smartphone or digital stethoscope. By employing deep learning, MySteth can classify heart murmurs with greater granularity, distinguishing between various types such as systolic, diastolic, Ejection Systolic Murmurs (ESM), and Pansystolic Murmurs (PSM), which have not been extensively categorized in previous studies. This technique effectively detects prevalent valvular heart conditions, including arrhythmia, mitral regurgitation, and coronary heart disease, at home. By leveraging widely available smartphones and digital stethoscopes, MySteth enhances cardiac diagnostics with precise, real-time analysis that is both accessible and cost-effective, marking a significant advancement in the field.

Recent innovations in wearable diagnostics, such as triboelectric sensors for arteriovenous fistula (AVF) monitoring in hemodialysis patients, highlight the growing feasibility of compact, non-invasive devices for continuous cardiovascular assessment (30). These systems, which utilize triboelectric impedance cardiography (T-ICG) to detect vascular abnormalities like stenosis through changes in signal morphology at key cardiac cycle points, underscore the clinical relevance of acoustic and impedance-based monitoring techniques. Inspired by such developments, our work aims to explore whether similar diagnostic precision can be achieved using more ubiquitous technology, namely, smartphones paired with digital stethoscopes and deep learning models. By enabling at-home screening of cardiac conditions through familiar devices, our approach complements and extends the paradigm of accessible, portable monitoring tools, especially in contexts where specialized equipment and expertise are limited.

The work by Galli et al. (31), which presents a portable, non-invasive ventilation (NIV) system for home and clinical use, offers a valuable reference model. Their device integrates airflow generation with pressure monitoring and remote smartphone-based data transmission, underscoring the importance of user-centered design and technological robustness in remote healthcare devices. For instance, recent advancements in wearable systems for arteriovenous (AV) fistula monitoring in dialysis patients have demonstrated the clinical feasibility and diagnostic value of portable acoustic sensing platforms (32). These systems use similar principles, capturing and analysing vascular sounds, to detect abnormalities such as stenosis, showcasing the real-world applicability of non-invasive auscultatory tools. Building upon such approaches, the MySteth system investigates whether commonly available devices like smartphones, when paired with digital stethoscopes and advanced deep learning models, can replicate and eventually democratize similar diagnostic capabilities.

## 2 Methods

**Figure 1:**
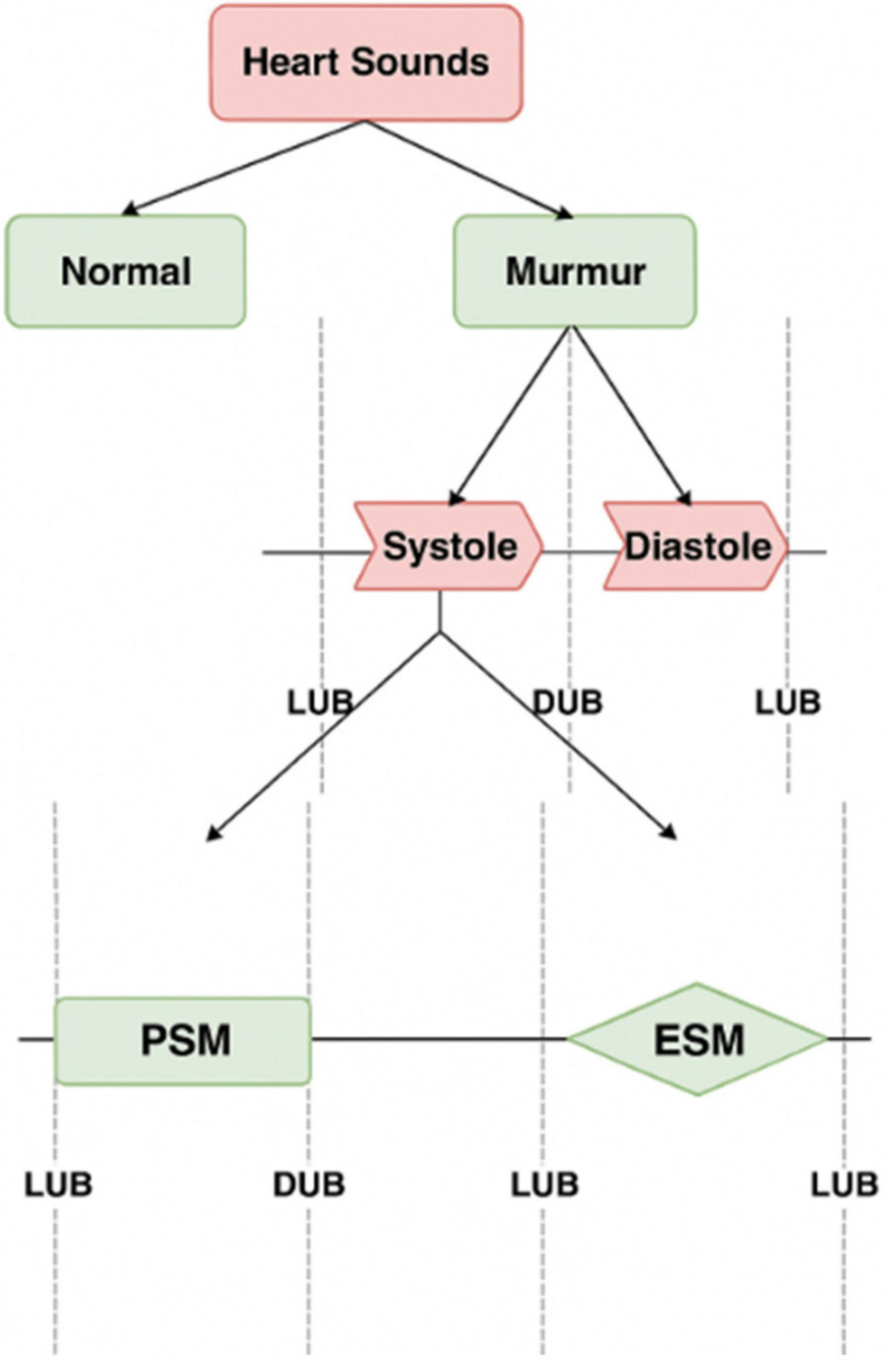
Classification of heart sounds into normal and murmurs, murmurs into systolic and diastolic murmurs, and further classifications to ESM and PSM.

In our work heartbeat is divided into two categories: murmurs, and normal heart sounds. We next divide the murmurs into systolic and diastolic murmurs. We further classify systolic murmurs into PSM and ESM. We don't need further categorization of diastolic murmurs, as most of the murmurs in this category are pathologic in nature and hence severe (33). The categorization shown in Figure 1 is the one identified by most of the doctors when they first examine a patient using a stethoscope. It is good enough to manifest evidence for a variety of heart disease. This procedure involves three classification steps to progressively refine the detection and categorization of heart sounds.

The complete procedure used to perform the classification in Mysteth is explained into two main parts: Data Preprocessing and the MySteth Architecture.

### 2.1 Data collection, labelling, preprocessing, refining, and data synthesis

**Figure 2:**
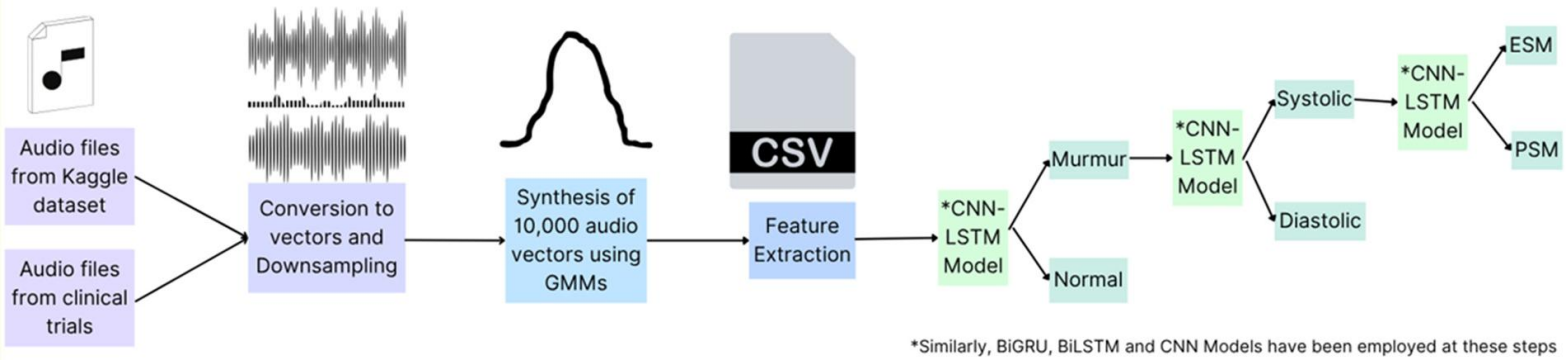
Preliminary steps for data preprocessing on the original dataset of 480 audio files selected. Created using Canva, licensed under Free Content License.

This part includes the steps shown in Figure 2, as explained below, which are applied on the original dataset to build a suitable Neural Network Model.

1. **Data Collection:** The authors used a publicly available Kaggle dataset (https://www.kaggle.com/kinguistics/heartbeat-sounds) to identify murmurs in heartbeat sound audios. The dataset contains 832 distinct heartbeats, of which 480 audios were selected for the use case. This dataset was gathered from the general public *via* the stethoscope Pro iPhone app and a clinic trial in hospitals using the digital stethoscope, DigiScope. In the original publicly available dataset of 832 heartbeat recordings, the distribution of samples was heavily skewed toward normal heart sounds, with murmurs forming a smaller proportion (only 129 audios). To avoid introducing bias from this imbalance, we selected 480 recordings that included 351 normal heartbeats and 129 murmurs, ensuring that both categories were adequately represented in the initial training set. This balanced selection was essential to prevent the model from underperforming on pathological cases.
2. **Data Labelling:** The publicly available dataset (https://www.kaggle.com/kinguistics/heartbeat-sounds) was annotated by Dr. Nishant Thakur, a super-specialized cardiologist from Max Hospital, I.P. Extension, Delhi, India, and re-annotated and cross-checked by Dr. Rajat Jain, a super-specialized cardiologist from Safdarjung Hospital, Delhi, India. Since the dataset is publicly available so no ethical approvals were required.
3. **Audio Processing and Refining:** Raw audios, sampled at 22050Hz, were down sampled to 4 kHz. This down sampling reduces computational load and storage requirements while retaining essential information for heartbeat analysis (34). Only the first 3 s of each audio were preserved to capture a complete cardiac cycle (S1 to S2 to S1), ensuring that the analysis encompasses all critical heart sounds. Audios shorter than 3 s were repeated to reach or exceed the 3-second duration, maintaining consistency in input length for the model. The study transformed audio signals into numerical data through the extraction of distinct features representative of signal characteristics, including amplitude, frequency, and duration, using the librosa library. Librosa is a widely-used Python library for audio analysis, known for its robust feature extraction capabilities, which facilitate effective signal characterization for subsequent classification (35).
4. **Data Synthesis:** Given the small initial dataset, Gaussian Mixture Models (GMM) were used to increase the dataset size to 10,000 audio vectors. This approach is beneficial as GMMs can generate new, realistic data points by modelling the probability distribution of the existing data, thus enhancing the dataset without additional data collection efforts (36). Out of the 10,000 vectors, 3,600 were murmurs, out of which 730 were systolic murmurs. GMMs were used again to increase the number of audio vectors represented by systolic murmurs to a size of 5,000. This targeted augmentation ensures that the dataset is well-balanced, particularly for the systolic murmur class, which is crucial for training a robust and unbiased classification model (37).

Specifically, the distribution of murmurs and normal heartbeats in the synthetic samples closely mirrors that of the original dataset, which consisted of 129 murmurs and 351 normal heartbeats recorded from a limited number of subjects—approximately 480 in total. Hence, it can be concluded that the total number of subjects for whom the data has been generated artificially is 9,520. The synthetic data was generated in a way that maintains subject-level diversity while amplifying underrepresented classes, particularly systolic murmurs. This alignment indicates that the GMM-based augmentation has effectively preserved the statistical properties and variability of the original dataset.

The use of data augmentation in scenarios with limited real-world samples is well-supported in literature; for instance, Frid-Adar et al. (38) demonstrated in synthetic data can significantly improve model performance when real data is scarce. Similarly, the targeted augmentation used here ensures the generation of high-quality, representative data, thereby enhancing model generalizability while reducing potential class imbalance. The proportion of heartbeats and murmurs, as well as its granular classifications in the generated dataset are similar to their proportions in the original dataset, thus suggesting that the data synthesis is appropriate and can be used for further experiments.

5. **Model Training:** Various models were trained on the refined datasets obtained from each of the following classification tasks. The train test ratios for all tasks were kept constant at a 70–30 percent split:
  a. Classification Task 1: Applied on the original dataset to separate the heartbeat sounds into normal heartbeats and murmurs
  b. Classification Task 2: Applied on the Murmurs obtained from classification task 1 to obtain systolic and diastolic murmurs
  c. Classification Task 3: Applied on the Systolic Murmurs obtained from classification task 2 to divide them into Pansystolic Murmurs (PSM) and Ejection Systolic Murmurs (ESM)

Various neural network models were applied on the dataset to obtain the best possible results:

1. CNN-LSTM:

The CNN-LSTM architecture was chosen due to its ability to effectively combine both spatial and temporal feature extraction, which is particularly important for the classification of heartbeat audio signals. CNNs can reduce noise by focusing on important features through convolutional filters, which makes the subsequent LSTM layers more effective in learning the temporal dependencies of the cleaned signal (39). Details of the models are as follows:

a. Input Layer: Processed numerical data representing the heartbeat audio signals.
b. Intermediate CNN and LSTM Layers, shown in Table 1.
c. Output Layer: SoftMax activation function to classify the audio signals into categories (e.g., normal heartbeat, murmur).

**Table 1:** Model architectures employed for training and testing on the audio vector and feature dataset.

S. No.	Model	Architecture
1	CNN-LSTM	Layer 1: CNN Layer with 9 filters, ReLU activation Layer 2: CNN Layer with 64 filters, ReLU activation Layer 3: CNN Layer with 32 filters, ReLU activation Layer 4: LSTM Layer with 8 neurons, tanh activation (default) Layer 5: LSTM Layer with 4 neurons, tanh activation (default)
2	BiLSTM	Layer 1: BiLSTM Layer with 128 neurons, tanh activation (default) Layer 2: BiLSTM Layer with 64 neurons, tanh activation (default) Layer 3: Dense Layer with 64 neurons, ReLU activation Layer 4: Dense Layer with 32 neurons, ReLU activation
3	CNN	Layer 1: CNN Layer with 9 filters, ReLU activation Layer 2: CNN Layer with 64 filters, ReLU activation Layer 3: CNN Layer with 32 filters, ReLU activation
4	BiGRU	Layer 1: BiGRU Layer with 128 neurons, tanh activation (default) Layer 2: BiGRU Layer with 64 neurons, tanh activation (default) Layer 3: Dense Layer with 64 neurons, ReLU activation Layer 4: Dense Layer with 32 neurons, ReLU activation

Furthermore, regularization methods were implemented in CNN-LSTM architecture to mitigate overfitting risks. A dropout of 20% was used in the LSTM layer, and the model was kept simple with a total of 5 layers.

2. BiLSTM: BiLSTMs have been successfully applied to various medical signal classification tasks, including ECG and phonocardiography (PCG) signals. Their effectiveness in capturing the temporal dynamics and dependencies in such data makes them a reliable choice for heartbeat classification (40). The details of the model are as follows:
  a. Input Layer: Processed numerical data representing the heartbeat audio signals.
  b. Intermediate BiLSTM and Dense Layers, shown in Table 1.
  c. Output Layer of Size 2 Units: SoftMax activation to classify the audio signals into categories (e.g., systolic murmur, diastolic murmur).
3. CNN: Heartbeat signals can exhibit significant variability in both time and frequency domains. CNNs, with their ability to apply convolutional filters across the input signal, can robustly handle such variations and capture essential characteristics of the heartbeat patterns (41). The details of the model are as follows:
  a. Input Layer: Processed numerical data representing the heartbeat audio signals.
  b. Intermediate CNN Layers, shown in Table 1.
  c. Output Layer: SoftMax activation function to provide classification probabilities. (e.g., Normal Heartbeat, Murmur)
4. BiGRU: Heartbeat signals are sequential in nature, and it is crucial to capture the temporal dependencies within the data. BiGRUs can process the input in both forward and backward directions, capturing dependencies from both past and future contexts, which is particularly beneficial for heartbeat classification (42). The details of the models are as follows:
  a. Input Layer: Processed numerical data representing the heartbeat audio signals.
  b. Intermediate BiLSTM and Dense Layers, shown in Table 1.
  c. Output Layer of Size 2 Units: SoftMax activation to classify the audio signals into categories (e.g., systolic murmur, diastolic murmur).

The results obtained from the models for each of the classification are articulated in the Results section. As the CNN-LSTM model gave the best accuracy scores, it was employed in the MySteth Architecture. This was followed by a SHAP (SHapley Additive exPlanations) on 10 test cases.

### 2.2 Mysteth architecture

**Figure 3:**
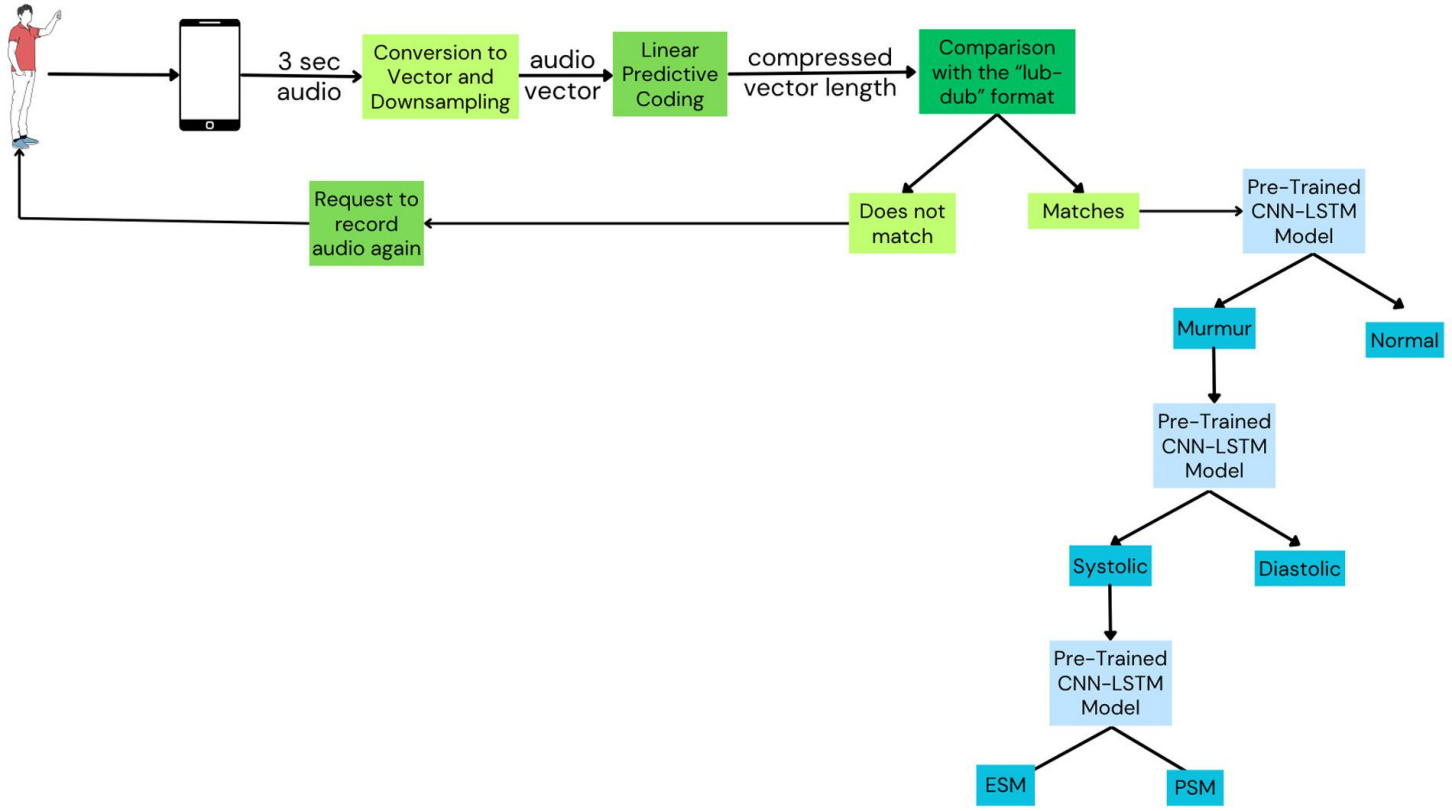
Mysteth architecture combining CNNs and LSTMs to classify heartbeats into its granular levels. Created using Canva, licensed under Free Content License.

The MySteth Architecture was designed to handle the refined datasets and perform the classifications at each step. The following steps, shown in Figure 3, are part of the processing pipeline:

1. **Recording:** A person records their heartbeat using a smartphone in a silent environment, capturing a 3-second audio clip.
2. **Down sampling and Compression:** Linear Predictive Coding (LPC) facilitates a two-step procedure of down sampling and compression of the recorded audio before training the models. By lowering the audio's sampling rate, down sampling effectively minimizes the amount of data and computing load while preserving crucial information. The spectral envelope of the digital voice signals is then compressed using LPC compression. By preserving important components of the heart sounds, this approach improves the efficacy of feature extraction (43). LPC minimizes the amount of data while preserving important information, which makes processing and analyzing the cardiac sounds simpler and quicker. In order to ensure that deep learning models can effectively capture and learn from the key elements of the heart sounds throughout the training phase, this preliminary step optimizes the data for the models (44). The integration of LPC for down sampling and compression in the preprocessing pipeline optimizes the data for deep learning models (45).
3. **Heartbeat Signal Verification:** The extracted features are compared to a reference model of a normal “lub-dub” heartbeat. The patient is asked to re-record the heartbeat if the signal does not match the classic “lub-dub” pattern.
4. **Model Training:** The audio vector and the extracted features passes through a pre-trained CNN-LSTM model that has shown the best results (as tabulated in Tables 1–3). This model had been trained previously on huge amounts of data (as explained in Part A). CNN and LSTM were applied serially due to the complementary nature of their roles in feature extraction and sequence modelling. The CNNs can preprocess and distil the essential features, which the LSTMs can then analyze in a temporal context (46).
  a. Static feature extraction is done by Convolutional Neural Networks (CNN).
  b. Temporal characteristics are extracted using Long Short-Term Memory (LSTM).
  c. Three convolution layers with kernel sizes of 9, 64, and 32 are applied, followed by batch normalization after each convolution layer. The details of the layers are given in Part A.
  d. For LSTM-based models, two layers of sizes 8 and 4 are added, followed by a dense layer. The details of the layers are given in Part A.
5. **Classification Task 1:** Two categories are created from the processed audio: murmur and normal heartbeat.
6. **Classification Task 2:** Murmurs are further divided into diastolic and systolic forms.
7. **Classification Task 3:** Systolic Murmurs are classified into Pansystolic Murmur (PSM) and Ejection Systolic Murmur (ESM).

**Table 2:** Evaluation metrics for different models for classifying heart sounds into normal, and murmur.

Model	Accuracy	Precision	Recall	F1 Score	ROC-AUC
BiLSTM	68%	0.68	1.00	0.81	0.50
CNN	72%	1.00	0.05	0.10	0.53
BiGRU	88%	0.98	0.17	0.30	0.59
CNN and LSTM	92%	0.73	0.93	0.82	0.93

**Table 3:** Evaluation metrics for different models for classifying murmurs into systolic murmur and diastolic murmur.

Model	Accuracy	Precision	Recall	F1 Score	ROC-AUC
CNN	68%	0.50	0.55	0.52	0.48
BiLSTM	72%	0.88	0.05	0.10	0.52
BiGRU	84%	0.65	0.95	0.77	0.70
CNN and LSTM	91%	0.80	0.70	0.75	0.83

## 3 Results

This section presents the results obtained from classifying heartbeat sounds using different models (as explained in Part A of the Methods section) into the following categories: Normal heartbeat, Murmurs, Systolic Murmur, Diastolic Murmur, PSM (Pansystolic Murmur), and ESM (Ejection Systolic Murmur).

**Figure 4:**
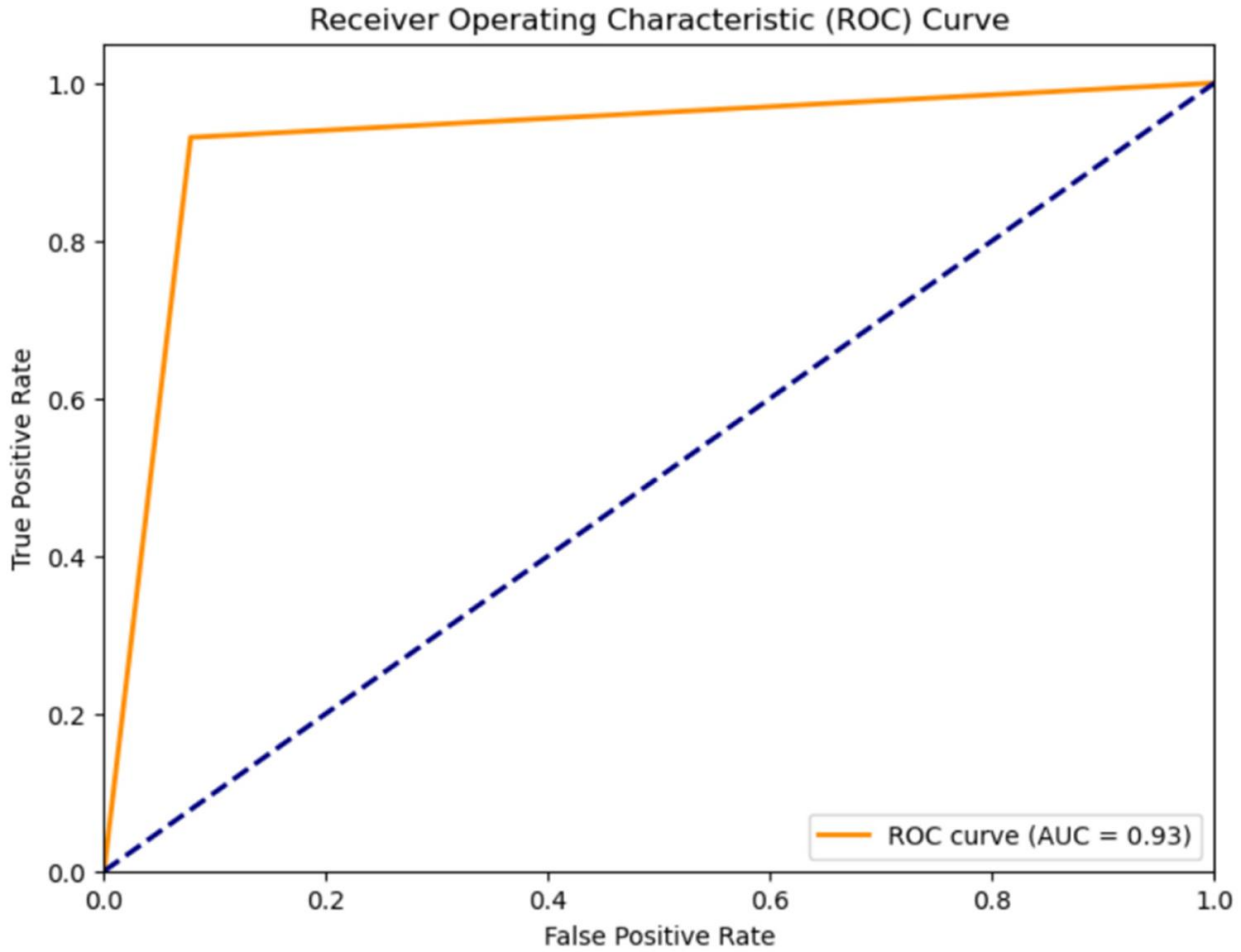
ROC curve for classification of heart sounds into normal and murmur using the CNN-LSTM model.

1. **Classification results for Normal Heartbeat, and Murmur:** The classification accuracy for Normal Heartbeat and Murmurs was evaluated using different models on the compressed audio representations obtained through Linear Predictive Coding (LPC). An overview of the outcomes, shown in Table 2, the Receiver Operating Characteristic Curve for the classification using the CNN-LSTM Hybrid Model, shown in Figure 4 justify the selection of a CNN-LSTM model.

The classification of heartbeat sounds is an essential task in the medical field, as it helps healthcare professionals diagnose various cardiovascular conditions. Although numerous research activities have been carried out to enhance the precision of heartbeat sound categorization, the majority of them have concentrated on refining data pre-processing methods or employing a single primary method such as neural networks, support vector machines, or hidden Markov models (47).

**Figure 5:**
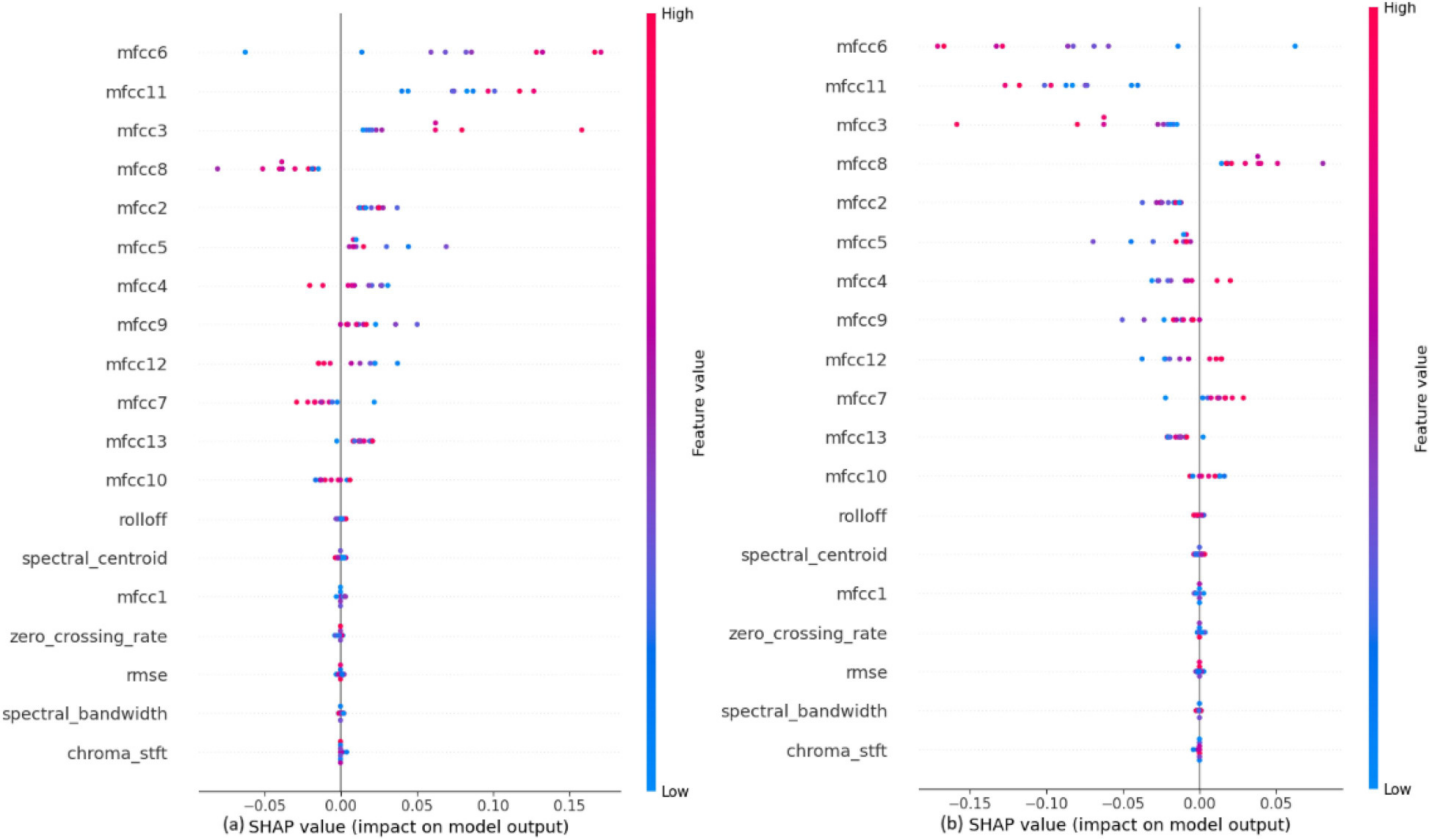
SHAP explanations for classifying heartbeat sounds into **(a)** normal heartbeat and **(b)** murmur.

The impact of each feature on the model, shown in Figure 5, to classify the heartbeat sounds into normal heartbeats or murmurs depicts that the MFCCs, specifically mfcc6, mfcc11, mfcc3 and mfcc8 have a considerable amount of weightage while making the predictions. It can also be concluded that the values of these features directly affect their SHAP values. For example, a high mfcc6 gives a highly positive SHAP value when predicting normal heartbeats, and a highly negative SHAP value when predicting murmurs. A similar trend is observed for almost all features. It is also noted that the features like chroma STFT, spectral bandwidth, RMSE and zero crossing rate have least impact on the model outputs.

**Figure 6:**
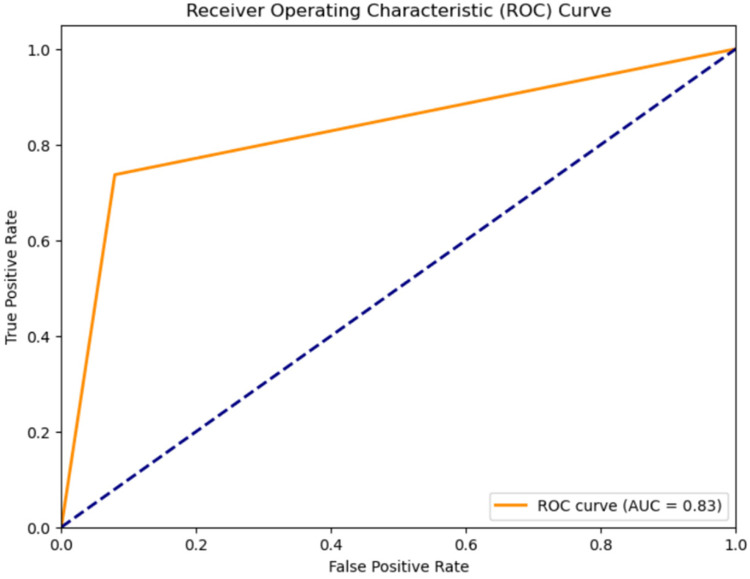
ROC curve for classification of murmurs into systolic murmurs and diastolic murmurs using the CNN-LSTM hybrid model.

2. **Classification results for systolic murmur, and diastolic murmur:** The classification accuracy for distinguishing between Systolic and Diastolic Murmurs was assessed using various models on the compressed representations of the murmur audio. The results, shown in Table 3, Receiver Operating Characteristic Curve for the classification using the CNN-LSTM Hybrid Model, shown in Figure 6 depict the efficiency of each model.

A hybrid classifier can significantly enhance classification accuracy. When combined in a hybrid CNN-LSTM model, these models can effectively extract deep features and contextual time data from Phonocardiogram (PCG) signals. The CNN component handles feature extraction, while the LSTM module extracts time-dependent features (48).

**Figure 7:**
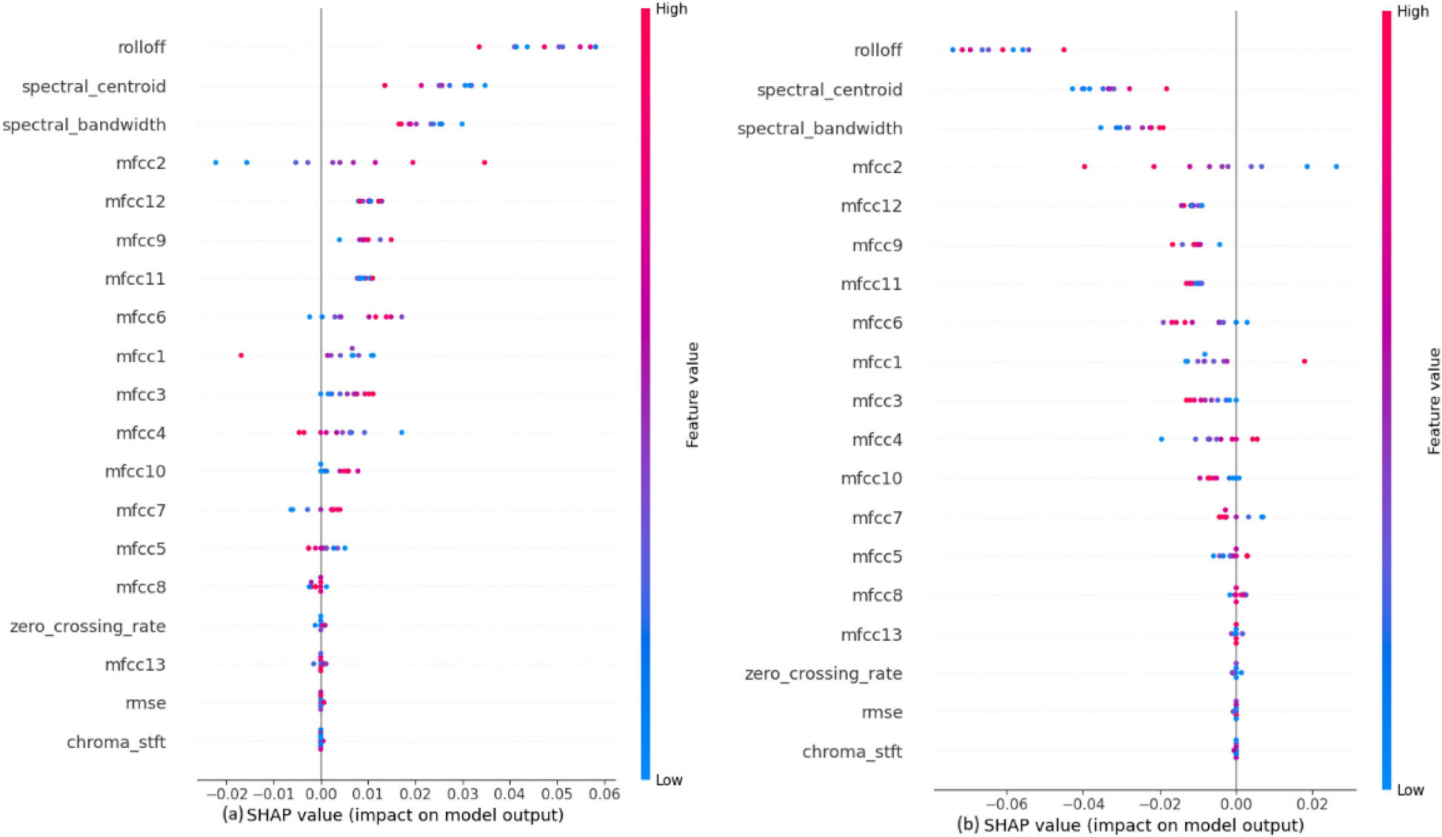
SHAP explanations for classifying murmurs into **(a)** systolic murmur and **(b)** diastolic murmur.

The impact of each feature on the model to classify the murmurs into systolic or diastolic murmurs, shown in Figure 7 depicts that the audio features like Rolloff, spectral centroid and spectral bandwidth have a considerable amount of weightage while making the predictions, which is contrary to the observation in the previous classification step. Among the MFCCs, mfcc2 seems to affect the model output the most. The values of these features directly affect their SHAP values. It is also noted that the features like chroma STFT, RMSE and zero crossing rate have least impact on the model outputs, just as in the previous classification step.

**Figure 8:**
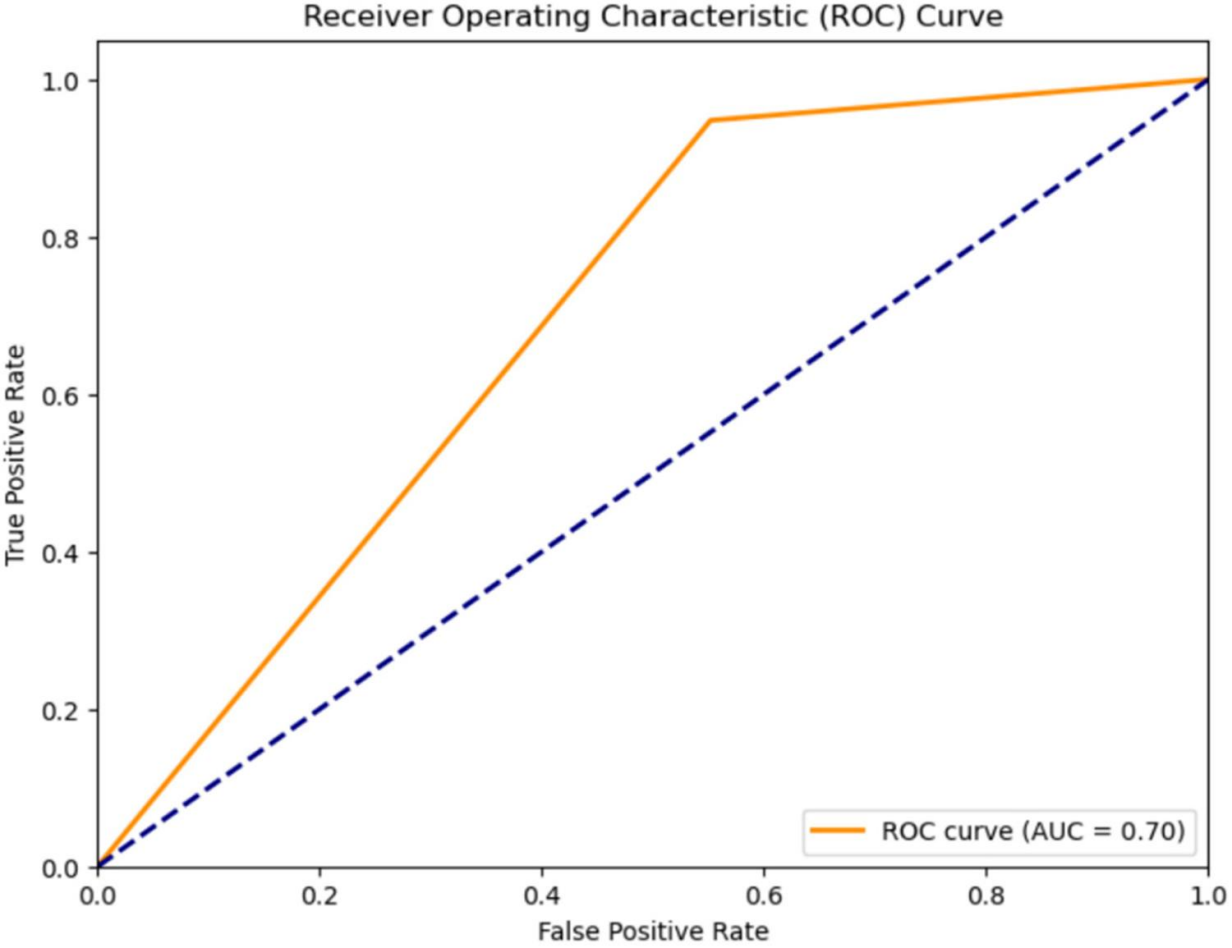
ROC curve for classification of diastolic murmurs into ESM and PSM using the CNN-LSTM hybrid model.

3. **Classification results for PSM, and ESM:** The accuracy for further classifying systolic murmurs into PSM and ESM was evaluated using various models on the compressed feature representations obtained from LPC feature extraction method. The outcomes, shown in Table 4, the Receiver Operating Characteristic Curve for the classification using the CNN-LSTM Hybrid Model, shown in Figure 8 justify the utility of the CNN-LSTM model.

**Table 4:** Evaluation metrics for different models for classifying systolic murmurs further into PSM, ESM.

Model	Accuracy	Precision	Recall	F1 Score	ROC-AUC
BiLSTM	51%	0.31	0.25	0.28	0.30
BiGRU	62%	0.43	0.42	0.41	0.36
CNN and LSTM	71%	0.60	0.54	0.56	0.54
CNN	90%	0.68	0.74	0.71	0.70

This hybrid approach has been shown to outperform single CNN or LSTM-based methods, producing richer and more concentrated models with higher performance and fewer parameters. These findings demonstrate how well different recurrent neural networks function in conjunction with convolutional neural networks to tackle challenging audio categorization problems. The utilization of LPC for feature extraction significantly contributes to the models' performance, especially in distinguishing subtle differences in heart sounds (49).

**Figure 9:**
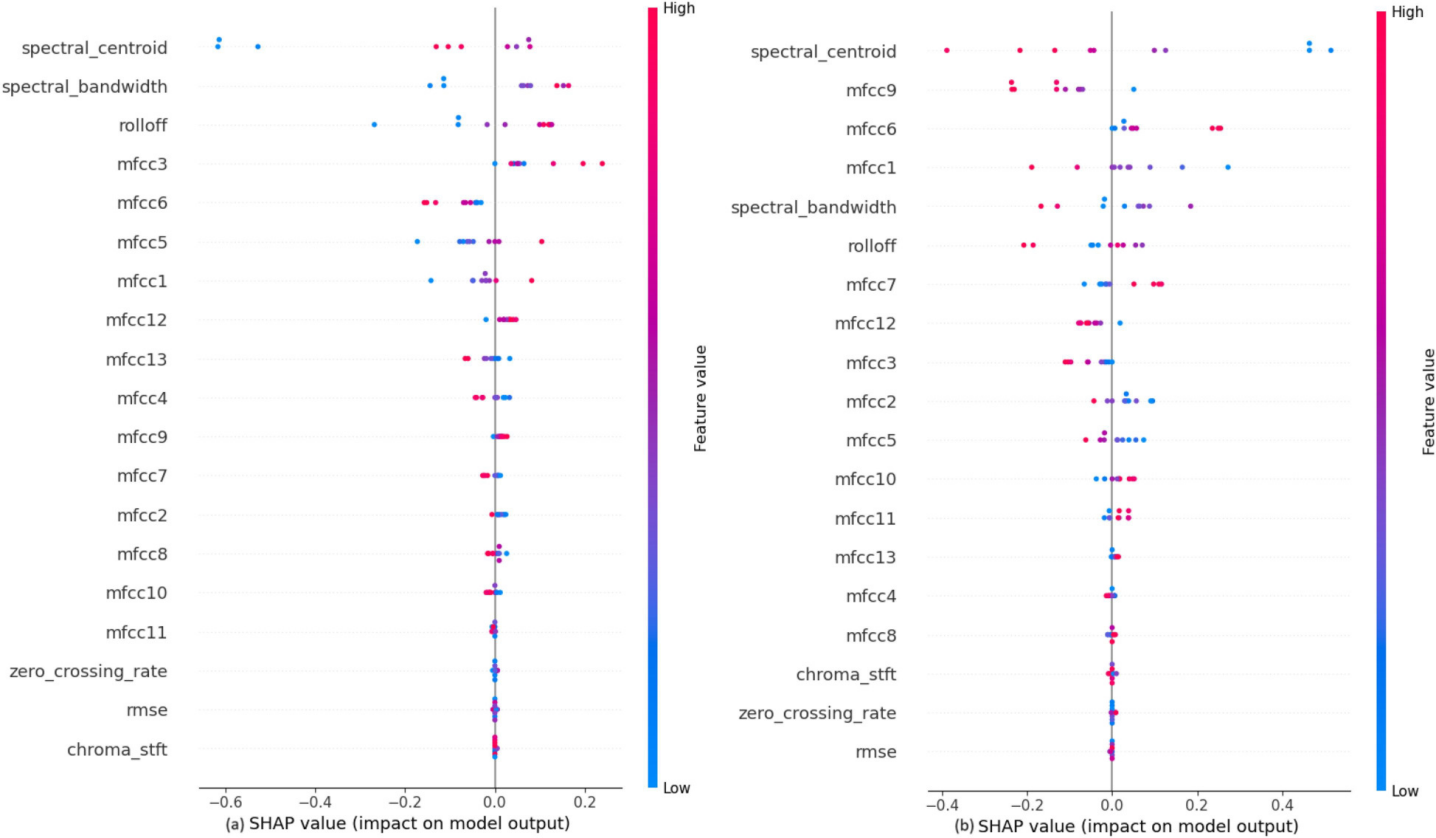
SHAP explanations for classifying diastolic murmurs into **(a)** ESM and **(b)** PSM.

The impact of each feature on the model to classify the systolic murmurs into ESM or PSM, shown in Figure 9, depicts that the audio features like Rolloff, spectral centroid and spectral bandwidth have a considerable amount of weightage while making the predictions, which is similar to the observation in the previous classification step. Among the MFCCs, mfcc3 seems to affect the model output the most. At this classification step, the values of these features do not affect their SHAP values, which is contrary to the previous two classification steps. It is also noted that the features like chroma STFT, RMSE and zero crossing rate have least impact on the model outputs, just as in the previous classification steps.

**Figure 10:**
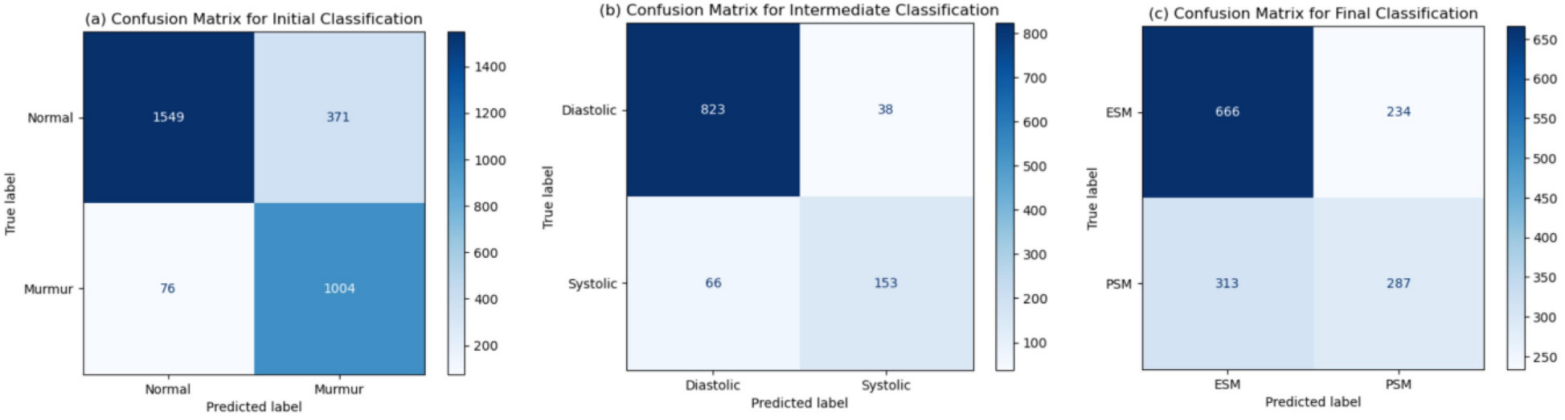
Confusion matrices obtained for the **(a)** initial, **(b)** intermediate, and **(c)** final classification tasks.

The statistical analysis, shown in Table 5, depicts the probable accuracy, precision and recall values obtained in a 95% confidence interval. These values are similar to the values obtained on the testing dataset, thus strengthening our results. Furthermore, statistical significance testing has been done against a random classifier. The null hypothesis states that the random classifier would be just as good as the model presented in the paper. The accuracy statistic is not very close, but the *p*-values are negligible for all classification tasks, signifying that our model performs much better than a random classifier baseline, hence disproving the null hypothesis. The confusion matrices for each classification task, shown in Figure 10, are representative of the metrics obtained. These have been reported for the testing dataset (whose size was around 3,000 for the initial, 1,080 for the intermediate, and 1,500 for the final classification tasks). A very important observation is that the number of heartbeats that are actually murmurs but classified as normal are very low [76 out of 3,000, as can be seen in Figure 10(a)]. This reiterated the fact that MySteth presents a reliable algorithm to be used for home-based heart health monitoring.

**Table 5:** Confidence intervals for evaluation metrics, and statistical significance testing for the model.

Classification task	Normal and Murmur	Systolic and diastolic	ESM and PSM
CI for accuracy	[0.910, 0.929]	[0.892, 0.926]	[0.884, 0.914]
CI for precision	[0.706, 0.753]	[0.739, 0.851]	[0.654, 0.712]
CI for recall	[0.913, 0.943]	[0.635, 0.756]	[0.710, 0.768]
Accuracy statistic	0.851	0.904	0.635
*p*-value	0.0	1.495 × 10^−178^	3.431 × 10^−26^

In conclusion, it has been established that the CNN-LSTM model out-performs the rest of the models when it is desired to classify heartbeats into higher granularity levels, while also giving insights into an interpretable decision-making process in order the predict the outcome at each classification step. While lightweight models such as shallow CNNs or simple feedforward networks may offer faster inference times, they lack the capacity to capture complex temporal patterns. Hence, the proposed CNN-LSTM model seems to be a good-fit in order to perform accurate classification while gaining the trust of medical professionals. This makes it a useful aspect of a home monitoring device, while decreasing the load on doctors.

## 4 Discussion

The introduction of MySteth as an innovative at-home heart diagnostic tool represents an advancement in the field of cardiac care, addressing critical gaps in the accessibility and convenience of preliminary heart investigations. This discussion focuses on the unique aspects and justifications for our approach, emphasizing the integration of CNN-LSTM architectures with Linear Predictive Coding (LPC) preprocessing, and the impact of these choices on the efficacy and practicality of MySteth.

The primary motivation for employing a hybrid CNN-LSTM model stems from the complementary strengths of Convolutional Neural Networks (CNNs) and Long Short-Term Memory networks (LSTMs) in handling the complexities of heartbeat sound classification (50). CNNs are adept at extracting spatial features from the input data, capturing local patterns and significant characteristics of the heart sounds. This capability is crucial for identifying the nuanced features present in heartbeat signals, such as murmurs and other anomalies. LSTMs, on the other hand, excel at modelling temporal dependencies and sequential patterns within the data. By integrating LSTMs with CNNs, we ensure that the model not only recognizes spatial features but also understands how these features evolve over time (51). This combination is particularly effective for analyzing heartbeat sounds, which inherently possess both spatial and temporal dimensions.

Linear Predictive Coding (LPC) plays a pivotal role in our approach by facilitating data compression and enhancing feature extraction. LPC reduces the complexity of the raw audio data while preserving essential information, making the subsequent processing by CNN and LSTM layers more efficient. This preprocessing step is crucial for improving the model's ability to detect subtle patterns and anomalies in the cardiac sounds, thereby enhancing classification accuracy (52). By incorporating LPC, we address the challenge of high data volume and computational load, enabling the use of advanced deep learning models even in resource-constrained environments. This efficiency is particularly beneficial for at-home diagnostic tools like MySteth, where minimal hardware requirements and quick processing are critical for user adoption and practicality (53).

Compared to conventional methods of heartbeat classification, which often rely on manual feature extraction and traditional machine learning algorithms, our CNN-LSTM approach offers several distinct advantages. Traditional methods can be limited by their dependency on handcrafted features and their inability to fully capture the complexity of the heartbeat signals. In contrast, deep learning models, particularly the CNN-LSTM combination, automatically learn relevant features from the data, leading to more accurate and robust classifications (54). Moreover, the ability to handle large and complex datasets without significant manual intervention makes our approach more scalable and adaptable to different healthcare settings. High accuracy rates have been achieved by our models, including the exceptional performance of the CNN-LSTM model with a 92% accuracy for classifying the heartbeats into normal and murmurs, and 91% for classifying the murmurs into systolic and diastolic murmurs. This underscores the effectiveness of our method in differentiating between normal and pathological heart sounds as well as finer distinctions such as various types of murmurs.

Unlike the recently developed hardware-centric designs (30–32), MySteth presents an end-to-end signal-processing using a deep learning model for heart sounds to perform segmentation and classification. This enables deployment on commodity devices and potentially broadening access where specialized sensors like PVDF triboelectric (30, 32) or NIV turbines (31) are impractical. Patient-cohort validations like B-/C-point slopes vs. stenosis (30, 32), and device-level performance envelopes (31) demonstrate physiologically interpretable trends. MySteth currently depends on SHAP explanations to justify the results of its analysis. Triboelectric ICG features (30, 32) have direct hemodynamic interpretations. However, MySteth uses algorithmic acoustic features, with less explicit references to mechanistic markers, such as the timing or the severity correlation, which may be a matter of concern for improving clinician trust to take triage decisions. These systems push the frontier in hemodialysis vascular monitoring (30, 32) and home respiratory therapy (31). MySteth advances acoustic cardiac screening on accessible hardware, that is a simple smartphone, thus filling a different but clinically adjacent niche that emphasizes software-driven auscultation and potential population-scale reach.

This work represents a pilot study aimed at evaluating the feasibility and effectiveness of deep learning–based heartbeat classification, and the results so far have been encouraging. The CNN-LSTM model demonstrated high accuracy in classifying normal and pathological heart sounds under controlled conditions, indicating strong potential for clinical relevance. While clinical validation has not yet been initiated, it is a key focus of our future work. While the manuscript does not explicitly analyze the impact of domestic ambient noise on system performance, it is important to note that domestic sounds—such as speech, appliances, or external traffic, have acoustic characteristics that are significantly different from those of heart sounds. These can be integrated as a preprocessing layer into the overall classification pipeline to improve robustness in real-world environments. Prior studies have demonstrated that heart sounds possess distinct acoustic characteristics, such as temporal regularity and specific frequency ranges, that differentiate them from other bodily sounds, making them suitable candidates for signal separation and denoising. Springer et al. (55) emphasized the effectiveness of frequency and envelope-based methods for isolating heart sounds from noise.

The current evaluation has been performed entirely on controlled and expert-annotated data. While this setup ensures high-quality ground truth for model development and benchmarking, it does not fully capture the variability, and recording artifacts present in real-world environments. Translation to home and clinical utility requires systematic prospective validation and robust noise-adaptation strategies. A prospective validation study can be conducted on diverse patient populations across different age groups, cardiac conditions, and recording environments. This study could include recordings from multiple devices (smartphones, digital stethoscopes) to assess hardware variability. Further, annotation of new data can be done correctly by multiple expert cardiologists to ensure reliable ground truth. Stratified sampling can also be done to capture a balanced representation of normal heart sounds and pathological murmurs, including rare subtypes.

The minimal hardware requirements and straightforward implementation of MySteth mean it can be readily adopted in various healthcare environments, including remote or under-resourced areas. This accessibility addresses a critical need in global healthcare, providing reliable and early detection tools for heart disease, which remains a leading cause of mortality worldwide (56). This paper distinguishes itself from previous research by introducing MySteth, a novel home-based heart monitoring tool that utilizes deep learning techniques to classify heart sounds with enhanced accuracy and detail. Unlike earlier studies that primarily concentrated on phonocardiography or ECG data—methods not easily accessible for home use—MySteth employs commonly available devices like smartphones and digital stethoscopes. It extends beyond basic heart sound classification by differentiating between normal heartbeats, murmurs, and specific subtypes such as Ejection Systolic Murmurs (ESM) and Pansystolic Murmurs (PSM). This level of granularity, particularly in home settings, has not been achieved by previous studies. The use of deep learning models for more precise and real-time analysis, combined with its accessibility and cost-effectiveness, marks this paper as a significant advancement in cardiac diagnostics over prior research.

This study focuses primarily on the development and evaluation of a pre-trained deep learning model for heartbeat classification, which can be used directly for real-time heartbeat classification. As the model is designed to be used as a pre-trained solution, the end user is not required to perform on-device training or intensive computation locally, hence nullifying the requirement of strong hardware for the same. Therefore, issues related to power consumption, battery life, and energy optimization strategies were not within the scope of this work, and need not be a cause of concern for the end-user. Finally, it is also noteworthy that the model predictions provide a good amount of interpretability using the SHAP values and help medical professionals gauge a better insight into the decision-making process, thus functioning as a helpful home heart-screening device for patients as well as a useful understanding of the prediction to the medical practitioners.

## 5 Future work

While our work presents significant advancements, there are limitations and areas for improvement. As already stated, not much study has been done in the area of categorizing mobile phone heartbeat sounds (57). To improve research in this area, a larger and more realistic dataset must be created (58). The models are trained and validated on specific datasets which may not encompass the full variability seen in global populations. Future work should focus on incorporating more diverse datasets to enhance generalizability. In this work, the audio was encoded using linear predictive coding. To compress audio, more encoding methods can be employed, such as auto-encoders. While our method is efficient, optimizing it further for real-time processing and deployment on portable devices could enhance its practical application. Future research should explore seamless integration with existing clinical workflows, ensuring that the technology is user-friendly for healthcare professionals. To confirm the models' long-term dependability and efficacy in practical situations, longitudinal research and comprehensive clinical trials are required (59).

The current study utilized uncleaned data recorded from smartphones, which included a significant amount of noise due to breathing artifacts. These artifacts can adversely affect the accuracy of classification. Future research could focus on removing these artifacts to enhance the signal-to-noise ratio, thereby improving the accuracy of murmur classification. Despite the presence of breathing artifacts in the current uncleaned data, the preliminary classification accuracies achieved were promising for screening purposes. This suggests that even without artifact removal, the current results may still offer valuable diagnostic information in a clinical setting. Further investigation is required to explore the potential of this technology as a tool for non-invasive heart screening, particularly in resource-limited settings where access to traditional cardiac diagnostics is limited.

Although domestic and breathing noise are recognized as important factors in real-world deployment, the present study did not include quantitative noise robustness experiments. The preprocessing pipeline can be adapted to better handle real-world acoustic conditions by incorporating adaptive band-pass filters, wavelet-based denoising, or spectral subtraction techniques to remove ambient and breathing artifacts without compromising clinically relevant heart sounds. Training can be made more robust by training with synthetically augmented datasets that include controlled levels of domestic noise (speech, TV, traffic, appliance hums, etc.), enabling the model to generalize better to variable acoustic environments. The utility can be improved by adding a pre-classification module that evaluates signal-to-noise ratio (SNR) and prompts re-recording if background noise is too high.

The deep learning model presented is relatively simple and could be enhanced by incorporating more complex features and deeper neural network architectures. By leveraging recent advances in deep learning, such as attention mechanisms and unsupervised feature learning, the classification accuracy could be further improved.

This work represents a preliminary investigation aimed at evaluating the feasibility and effectiveness of deep learning–based heartbeat classification, and the results so far have been encouraging. The CNN-LSTM model demonstrated high accuracy in classifying normal and pathological heart sounds under controlled conditions, indicating strong potential for clinical relevance. The next step involves testing the system in real-world settings, including diverse patient populations and varied acoustic environments, such as outpatient clinics and home-based monitoring.

The current study primarily utilizes synthetic data generated through a Gaussian Mixture Model (GMM)-based augmentation approach, which was designed to preserve subject-level diversity while amplifying underrepresented classes, particularly systolic murmurs. This method ensures that the augmented dataset maintains the statistical properties and variability of the original recordings, thereby supporting robust model training. To address generalizability beyond the training data, future work will focus on collecting and evaluating data from real-world use of the MySteth prototype in diverse clinical and home environments.

In conclusion, this preliminary work lays the groundwork for future efforts to enhance heartbeat classification accuracy using smartphone recordings. Proposed future directions include removing noise artifacts from the data, employing more sophisticated deep learning models, expanding the classification scope, and collecting larger datasets. Ultimately, these advancements will refine murmur detection and improve the quality of cardiac diagnostics provided by smartphone recordings.

## 6 Conclusions

MySteth is a tool that our study introduces. Using deep learning algorithms and just the sound of a heartbeat recorded using a phone or digital stethoscope, the authors investigated the screening of a broad class of heart disorders. Heartbeats can be categorized by MySteth into three categories: normal, systolic, and diastolic murmurs. Systolic murmurs can also be further classified into two categories: Ejection Systolic Murmur (ESM) and Pansystolic Murmur (PSM).

In order to keep the condition from getting worse to the point where it becomes fatal or irreversible, this effort can be very helpful in identifying the onset of a wide class of cardiovascular heart diseases (60). MySteth, a tool in the field of heart sound classification, can significantly contribute to preventive healthcare and lower the total burden of cardiovascular illnesses by enabling at-home early screening and precise diagnosis of heart murmurs and other irregularities. Because of its architecture, the instrument can be deployed in a variety of contexts, such as remote and rural locations, allowing disadvantaged groups to benefit from modern diagnostics. The scalable and adaptable nature of MySteth ensures it can be integrated into different healthcare environments, from large urban hospitals to small rural clinics and even home-based care. By addressing both the technological and practical challenges in this domain, MySteth stands out as a viable solution with significant potential for improving global health outcomes.

## Data Availability

Publicly available datasets were analyzed in this study. This data can be found here: https://www.kaggle.com/kinguistics/heartbeat-sounds.
